# The plant stem-cell niche and pluripotency: 15 years of an epigenetic perspective

**DOI:** 10.3389/fpls.2022.1018559

**Published:** 2022-10-31

**Authors:** Ralf Müller-Xing, Qian Xing

**Affiliations:** Lushan Botanical Garden, Chinese Academy of Sciences, Jiujiang, China

**Keywords:** stem-cell formation and determinacy, pluripotent stem-cell lineages, callus formation, adventitious shoots and roots, pluripotency factors, epigenetic regulators, chromatin remodeling and modifications

## Abstract

Pluripotent stem-cells are slowly dividing cells giving rise to daughter cells that can either differentiate to new tissues and organs, or remain stem-cells. In plants, stem-cells are located in specific niches of the shoot and root apical meristems (SAMs and RAMs). After ablation of stem-cell niches, pluripotent meristematic cells can establish new stem-cells, whereas the removal of the whole meristem destructs the regeneration process. In tissue cultures, after detached plant organs are transferred to rooting or callus induction medium (G5 or CIM), vasculature-associated pluripotent cells (VPCs) immediately start proliferation to form adventitious roots or callus, respectively, while other cell types of the organ explants basically play no part in the process. Hence, in contrast to the widely-held assumption that all plant cells have the ability to reproduce a complete organism, only few cell types are pluripotent in practice, raising the question how pluripotent stem-cells differ from differentiated cells. It is now clear that, in addition to gene regulatory networks of pluripotency factors and phytohormone signaling, epigenetics play a crucial role in initiation, maintenance and determination of plant stem-cells. Although, more and more epigenetic regulators have been shown to control plant stem-cell fate, only a few studies demonstrate how they are recruited and how they change the chromatin structure and transcriptional regulation of pluripotency factors. Here, we highlight recent breakthroughs but also revisited classical studies of epigenetic regulation and chromatin dynamics of plant stem-cells and their pluripotent precursor-cells, and point out open questions and future directions.

## Introduction

Unlike animals, plant growth and organ formation occur post-embryonically, mediated by meristems that are located on the tips of growth axes in shoots and roots (Doerner, 2003). Meristems contain a specialized cellular microenvironment known as stem-cell niche (SCN) that provides the signals and physical support to maintain the pluripotent stem-cells (Sablowski, 2011). The SCN is surrounded by a transitory population of indeterminate cells that give rise to determinate cells and organs. Shoot and root apical meristem (SAM and RAM), which are formed during embryogenesis, only contributes to the main stem and main root, respectively. Branched structures rise post-embryonically from secondary meristems initiated from a few cells that retain meristematic characteristics (Nicolas and Laufs, 2022). In SAMs, the stem-cells, located at the top of the meristematic dome, secret the signal peptide CLAVATA3 (CLV3) that represses the pluripotency gene *WUSCHEL* (*WUS*) in cells of the organizing center (OC) underneath the stem-cells (Müller-Xing and Xing, 2021). Along with the pluripotency factor SHOOT MERISTEMLESS (STM), WUS maintains the stem-cells that form with the OC the shoot SCN. RAMs include mitotically less active organizer cells called the quiescent center (QC) and the surrounding initials, which together compose the root SCN. Similar to WUS, the pluripotency factor WOX5 maintains the stemness of the initials (Sarkar et al., 2007). Hence, ‘organizing’ cells maintain the stem-cells and SCNs by continuous short-range signaling. In 2007, Ben Scheres proposed in his landmark review that the SCNs of plant and animal kingdoms are specified by kingdom-specific patterning mechanisms, but that connect to a related core of epigenetic stem-cell factors (Scheres, 2007). Growing evidence endorses that plant stem-cell fate is also determined by epigenetic mechanisms, since the first models for an epigenetic control of plant SCNs were proposed 15 years ago.

**Figure 1:**
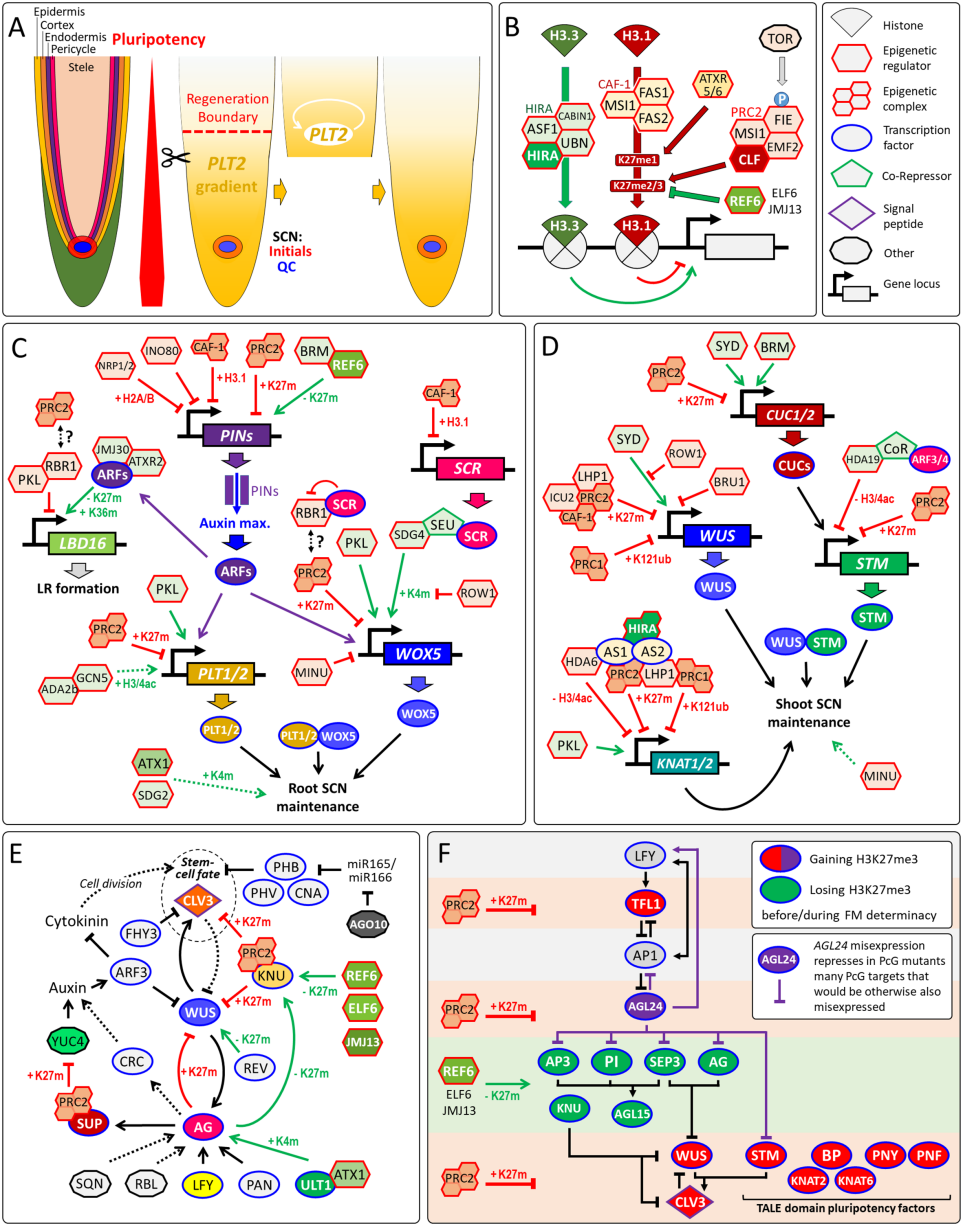
Regeneration competence of RAMs and epigenetic regulation of pluripotency genes. **(A)** Organization of the RAM and dependency of root tip regeneration on the pluripotency factor PLT2. After resection, *PLT2* confers regeneration potential to differentiating cells *via* auto-activation. Notably, resection beyond the regeneration boundary prevents regeneration of the SCN. **(B)** Epigenetic gene regulation by HIRA, CAF-1, PRC2 and REF6. Beside the eponymous histone chaperone, the HIRA complex contains Anti Silencing Factor 1 (ASF1), Calcineurin Binding protein 1 (CABIN1) and Ubinuclein (UBN) 1/2 (Nie et al., 2014), while FASCIATA1/2 (FAS1/2) and MULTICOPY SUPPRESSOR OF IRA1 (MSI1) are subunits of CAF-1 complex (Kaya et al., 2001). The demethylase REF6 can interact with BRM and bind sequence-specifically its target genes to remove H3K27me3 (Li et al., 2016). **(C)** Direct and indirect epigenetic control of root pluripotency factors. Removal of H3K27me3 by REF6 is required to maintain PIN1/3/7 expression, while PRC2 represses *PIN1* by H3K27me3 (Gu et al., 2014; Wang et al., 2019). Note that altered PIN expression indirectly changes the expression of pluripotency genes *via* AUXIN RESPONSE FACTORs (ARFs). ARF7/19 recruits H3K27me3-demethylase JMJ30 and H3K36me3-transferase ATXR2 to activate *LATERAL ORGAN BOUNDARIES DOMAIN16/29* (*LBD16/29*) that encode key regulators of lateral root (LR) formation, while PKL recruits RBR1 that represses *LBD16* (Lee et al., 2018a; Lee et al., 2018b; Ötvös et al., 2021). ROW1 specifically binds H3K4me3 at the *WOX5* promoter to repress its transcription (Zhang et al., 2015). General Control Nonderepressible protein5 (GCN5), and its activator ADA2b promote *PLT1/2* expression (Kornet and Scheres, 2009). Note that most pluripotency factors are PcG/H3K27me3-targets (Zhang et al., 2007; Shu et al., 2019). Hence, global changes of H3K27me3 could affect their expression directly and indirectly *via* changed auxin signaling by altered PIN levels. RBR1 interacts with the PRC2 component FIE (Mosquna et al., 2004), but it remains unclear whether this connection is relevant for the GRN of the root SCN. **(D)** Maintenance of the shoot SCN by epigenetic regulation of root pluripotency factors. **(E)** Floral stem-cell determinacy through silencing of *WUS* by a complex GRN (Reviewed in (Shang et al., 2019). During flower stage 2, LEAFY (LFY) and WUS as well as the TrxG proteins ULT1 and ATX1 activate *AG* that in turn activate *SUPERMAN* (*SUP*) and *KNUCKLES* (*KNU*). In flower stage 6, KNU represses *CLV3* and *WUS* (Shang et al., 2021). Many of these transcriptional regulations are supported or driven by changes in H3K4me3 (K4m) or H3K27me3 (K27m), which is also confirmed by the flower indeterminacy phenotypes by loss of H3K27me3 demethylases REF6, ELF6 and JMJ13 or PRC2 (Yan et al., 2018; Müller-Xing et al., 2022). Other genes directly or indirectly involved in *WUS* silencing: ARGONAUTE1 (AGO1), AUXIN RESPONSE TRANSCRIPTION FACTOR3 (ARF3/ETT), CRABS CLAW (CRC), FAR-RED ELONGATED HYPOCOTYLS3 (FHY3), PERIANTHIA (PAN), REBELOTE (RBL), SQUINT (SQN), YUCCA4 (YUC4) and the HD-ZIP class III transcription factors CORONA (CNA), PHABULOSA (PHB), PHAVOLUTA (PHV) and REVOLUTA (REV). **(F)** GRN of floral stem-cell determinacy sustained by changes in H3K27me3 levels at gene loci of pluripotency genes and other key regulators (after (Müller-Xing et al., 2022), modified). STM enhances binding of WUS to the *CLV3* chromatin through STM-WUS heterodimerization (Su et al., 2020). Arrows, transcriptional activation. Arrows with blunt ends represent repression. Double-sided arrows, protein-protein interaction. Dotted arrows indicate either indirect regulation or possible direct regulation that is not yet verified. Arrows in green indicate positive epigenetic regulation, in red, negative epigenetic regulation, and in black, non-epigenetic regulation. K4m, H3K4me3; K27m, H3K27me3; K36m, H3K36me3; H3/4ac, H3/H4 acetylation.

High regeneration capacity is a feature of plant development. After loss of the SCN by laser ablation, the pluripotent meristematic cells can establish new stem-cells in SAMs and RAMs (Reinhardt et al., 2003; Xu et al., 2006). The removal of whole meristems leads to destruction of the regeneration process (Sachs, 1994), indicating that meristematic cells are more pluripotent than somatic cells. In the RAM, the stem-cell regeneration competence correlates with the expression gradient of pluripotency factor PLETHORA2 (PLT2) (**Figure 1A**) (Durgaprasad et al., 2019). There are at least two ways how epigenetic regulators control stem-cell fate: (i) they directly regulate the gene loci of pluripotency factors; or (ii) they regulate genes that are required to maintain the meristem organization that indirectly preserves the SCN, this may include indirect regulation *via* phytohormone pathways. For instance, altered levels of the repressive histone mark H3K27me3 at *PIN* gene loci, which encode Auxin efflux-carriers, results in altered auxin gradients, RAM size and lateral root primordia (LRP) numbers (**Figure 1C**). This perspective focuses on the epigenetic regulation of pluripotency genes that control stem-cell fate in *Arabidopsis thaliana*.

## Direct and indirect regulation of pluripotency factors by epigenetic chromatin modifiers and remodelers in the RAM

Epigenetic gene regulation bases on post-translational histone modifications, nucleosome assembly and ATP-dependent chromatin remodeling, which controls the accessibility of chromatin to transcription factors. Although both chaperone complexes target histone H3 variants, Histone Regulator A (HIRA) complex and chromatin assembly factor-1 (CAF-1) complex have opposite effects on epigenetic gene regulation (**Figure 1B**). The HIRA complex deposits H3.3, which facilitates transcription, in a DNA synthesis-independent manner (Nie et al., 2014), whereas H3.1 deposition, which is essential for maintenance of the repressive H3K27me3 mark through cell division, relies on the heterotrimeric CAF-1 complex (Jiang and Berger, 2017). CAF-1 maintains cellular and functional organization and expression of the pluripotency gene SCARECROW (SCR) in the RAM (Kaya et al., 2001). CAF-1 and the H2A/H2B histone chaperone NAP1-RELATED PROTEIN1/2 (NRP1/2) play synergistic roles in root SCN maintenance by rather maintaining of the auxin gradient maximum at the QC than directly controlling the expression of pluripotency genes such as *WOX5* and *PLT1* (Ma et al., 2018). Similarly, NRP1/2 and the chromatin-remodeling factor INOSITOL AUXOTROPHY 80 (INO80) synergistically maintain histone H3 levels within the chromatin regions of *PIN1*, while the simultaneous loss of these three genes results in higher PIN1 protein levels, perturbed auxin gradients and misexpression of the root pluripotency factors WOX5 and PLT1/2 (Kang et al., 2019). On the contrary, the SWI2/SNF2-family chromatin-remodeling factor BRAHMA (BRM) positively regulates the expression of several *PINs* as well as *PLT1/2*, but ChIP experiments indicate that only the *PIN* genes are directly targeted by BRM (Yang et al., 2015). Hence, loss of *BRM* affects the expression of *PLT1/2* rather indirectly through impaired auxin signaling by reduced *PIN* expression levels. In contrast, the non-canonical SWI2/SNF2-type ATPase MINUSCULE2 (MINU2) directly activates the promoter of *WOX5* (Sang et al., 2012).

The repressive Polycomb group (PcG) proteins and the activation-related Trithorax group (TrxG) proteins have been implicated to regulate SCN maintenance and meristem activity (Singh et al., 2020). The Polycomb Repressive Complex 2 (PRC2) deposits the repressive H3K27me3 mark, while PRC1 sets the repressive H2AK121ub mark independently of H3K27me3 (Zhou et al., 2017). TrxG proteins were defined as their antagonists that can range from chromatin-remodelers to histone modifiers that deposit activation-related marks such as H3K4me3 and histone acetylation (Müller-Xing et al., 2014b).

A key role of PRC1 is the repression of several key regulators such as *WOX5* and *PLT1/2* controlling root SCN specification and cell proliferation (Merini et al., 2017). The CHD3-type chromatin-remodeler PICKLE (PKL) is a TrxG protein and acts antagonistically to the PcG protein and H3K27me3-transferase CURLY LEAF (CLF) controlling RAM activity (Aichinger et al., 2011). Altered meristematic activity in *pkl* and *clf* mutants correlates with changed H3K27me3 levels and altered expression of these pluripotency genes (Aichinger et al., 2011). Loss of PKL decreases meristematic activity with an increased H3K27me3 level at *WOX5* and *PLT1/2*, whereas mutation in *CLF* increases meristematic activity of the root with loss of the H3K27me3 level. In line with the negative effect of H3K27me3 on root pluripotency genes and SCN, the H3K4-histone methyltransferases ARABIDOPSIS HOMOLOG of TRITHORAX1 (ATX1/SDG27) and SET DOMAIN GROUP2 (SDG2) promote the root SCN integrity (Yao et al., 2013; Napsucialy-Mendivil et al., 2014), but it is not yet known whether ATX1 and SDG2 directly bind to the chromatin of pluripotency genes.

Most chromatin modifiers and remodelers do not possess a DNA-binding domain that would allow sequence-specific binding to their target genes. Hence, one of the recruitment strategies of epigenetic regulators such as PRC2 is through intermediary transcription factors, which facilitate recruitment to the target chromatin (Godwin and Farrona, 2022). Notably, pluripotency factors can be such recruiters of epigenetic regulators. WOX5, which is expressed in the QC, functioning as a mobile organizer signal that represses differentiation in neighboring columella stem-cells. There, WOX5 recruits the co-repressors TPL/TPRs that, in turn, recruit histone deacetylase HDA19 to silence the differentiation factor *CDF4 via* histone deacetylation (Pi et al., 2015). Recently, it has been implied that the EAR-domain of TPL can also recruit PRC2-activity, but the proof of a direct TPL-PRC2 interaction is pending (Baile et al., 2021). *WOX5* transcription is also epigenetically regulated. SCR recruits SEUSS (SEU), a homologue of the animal LIM-domain binding (LDB) proteins, to the *WOX5* promoter. Subsequently, SEU recruits the methyltransferase SDG4 that deposits H3K4me3 at the *WOX5* promoter activating this key pluripotency factor in the RAM (Zhai et al., 2020).

RETINOBLASTOMA-RELATED1 (RBR1) is a master regulator of the cell cycle and root development. In 2007, Ben Scheres suggested RBR1 as one potential link between stem-cell regulation and chromatin modifications in plants (Scheres, 2007). This assumption largely relied on a study in mammalian research revealing that retinoblastoma (RB) protein targets PRC2 to the promoter of cell-cycle control genes (Kotake et al., 2007). Since, it has be shown that RBR1 is required for silencing of late embryonic genes by increasing H3K27me3 levels *via* PRC2 in plants (Gutzat et al., 2011). Several studies deepened our knowledge about how RBR control RAM development (reviewed in (Desvoyes and Gutierrez, 2020) but elucidation of a potential RBR1-PRC2 pathway involved in controlling the root SCN awaits further research.

## Epigenetic regulation of the shoot SCN, a TALE of activation and silencing

In retrospect, the article of (Takeda et al., 2004) about TONSOKU (TSK/BRUSHY1/MGOUN3) can be considered as one of the first studies that addressed the role of epigenetics in maintaining the shoot SCN. The authors showed that loss of *TSK* results in disorganized SAMs with an abnormal, dispersed *WUS* expression pattern, and suggested that TSK links DNA-repair and epigenetic gene silencing, but the exact molecular function of TSK remained vague. A most recent study revealed that TSK-mediated DNA-repair, which can rescue broken DNA-replication forks, involves specific interaction of TSK with H3.1 *via* recognition of alanine 31 (Davarinejad et al., 2022). H3.1 is essential for maintenance of H3K27me3 by PRC2 through cell division and silencing of the TALE class I *KNOX* and pluripotency genes *STM*, *BP*/*KNAT1*, *KNAT2*, and *KNAT6* (Jiang and Berger, 2017). Hence, the ability of TSK to distinguish H3.1 from other H3 variants might play a role in TSK-dependent epigenetic gene repression and should be addressed in future studies.

SWI2/SNF2-type chromatin-remodelers, such as BRM and its homologue SPLAYED (SYD), play an important role in the accessibility of cis-regulatory DNA regions to transcription factors. In SAMs, SYD is recruited to the *WUS* promoter and directly activates *WUS* transcription, which is involved in maintenance of the shoot SCN (Kwon et al., 2005). The recruiter of SYD to the *WUS* chromatin is not yet known and is a question to be addressed. BRM and SYD are not likely to activate *STM* directly but *via* direct activation of *CUC* genes (Kwon et al., 2006). The PHD-domain protein REPRESSOR OF WUSCHEL1 (ROW1/BARD1) confines *WUS* expression into the OC by binding to the *WUS* promoter near the SYD-binding site. Since ROW1 and SYD interact in Co-IP assays, BARD1 may repress *WUS* transcription *via* inhibition of SYD-dependent chromatin-remodelling that activates *WUS* expression (Han et al., 2008; Han and Zhu, 2009). Also two non-canonical SWI2/SNF2-type ATPases MINU1/MINU2 are essential to maintain the shoot SCN but do not bind to the *WUS* chromatin (Sang et al., 2012).

Strongly depleted PRC2 function results in enlarged SAMs with increased *STM* expression levels and expanded expression domain of the stem-cell marker *CLV3*, while *WUS* expression and domain size were decreased, indicating an uncoupling of stem-cell fate and *WUS* expression levels (Müller-Xing et al., 2014a; Müller-Xing et al., 2015; Müller-Xing et al., 2022). This downregulation of *WUS* might be caused by upregulation of *WUS* repressors such as KNU that is an H3K27me3-target. The mRNAs of *STM* and *KNAT1* are expressed in the SAM but are silent in leaf and early stages of flower primordia (Lincoln et al., 1994; Long et al., 1996). The ASYMMETRIC LEAVES1 (AS1)-AS2 transcription factor complex recruits PRC1, PRC2 and the PcG-associated protein LHP1 to chromatin of *KNAT1*, *KNAT2*, and *KNAT6* to silence these pluripotency genes (Lodha et al., 2013; Z. Li et al., 2016). Similarly, ARF3/4 silence directly *STM via* histone-deacetylation in flower primordia, although the co-repressor, which links ARF3/4 with HDA19, still waits to be discovered (Chung et al., 2019). This strong epigenetic repression of pluripotency genes in organ primordia and differentiated tissue leads to the question how the stem-cells of auxiliary (AMs) and flower meristems (FMs) can be *de novo* initiated.

## Epigenetically maintained pluripotency facilitates *de novo* stem-cell formation during shoot branching

**Figure 2:**
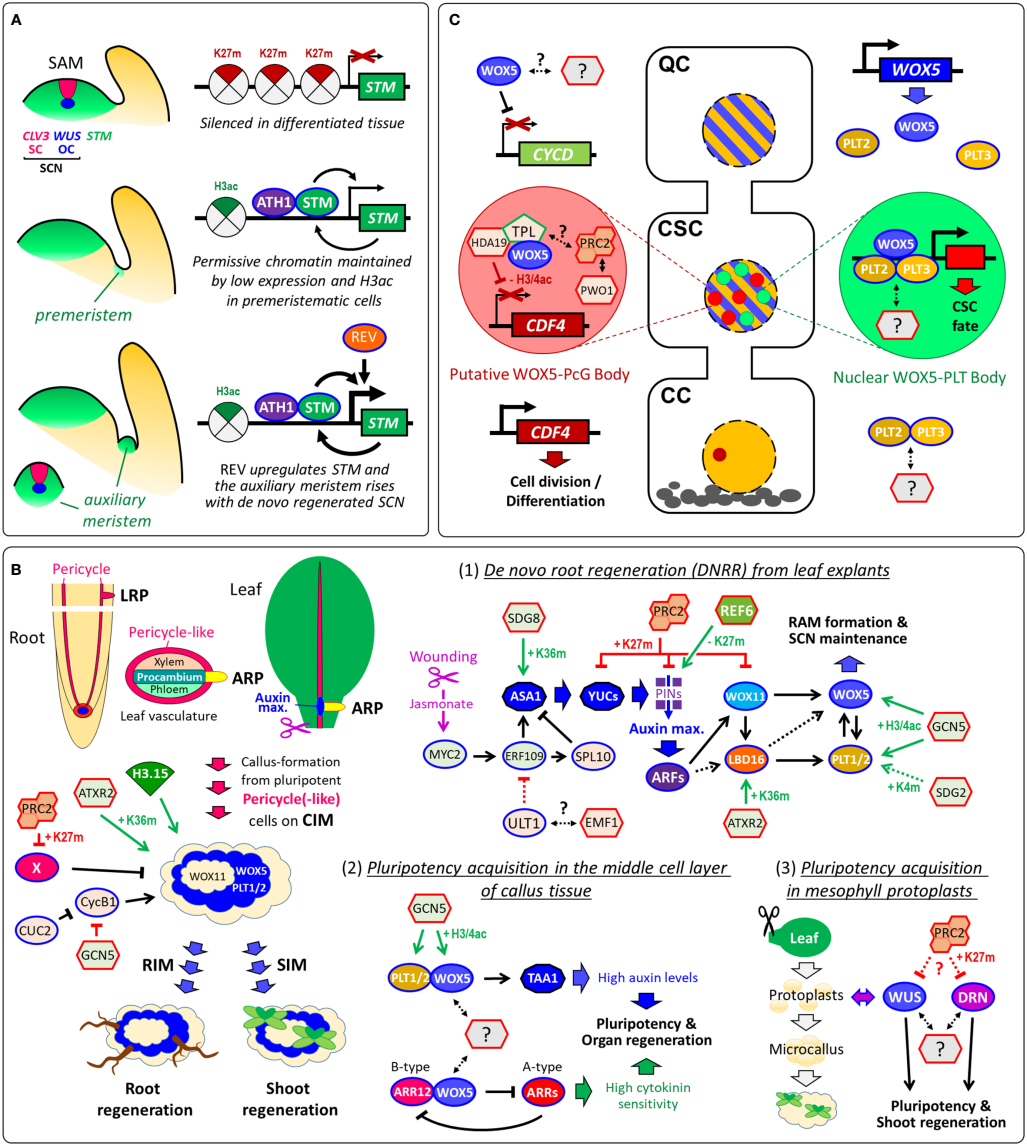
Pluripotency acquisition during *de novo* stem-cell formation and *de novo* organogenesis. **(A)** Organization of the SAM and epigenetic maintenance of premeristem cells for shoot branching. *STM* is silenced by H3K27me3 in differentiated tissue, while low expression rates of STM, which interacts with ATH1, auto-activates its own transcription in premeristemic cells. REV directly upregulates *STM* expression in leaf axil meristematic cells (Shi et al., 2016). **(B)** Vascular-associated pluripotent cells are key for callus formation and *de novo* organogenesis. Several epigenetic regulators control callus development including PRC2 that promotes callus formation by repressing leaf-regulatory genes (X) by H3K27me3 (He et al., 2012). Although CUC2 and GCN5 promote shoot regeneration, both suppress M/G2-phase marker *CYCB1;1* and cell proliferation in callus tissue (Daimon et al., 2003; Kim et al., 2018; Li et al., 2016). (1) Conceptional model of the transcriptional and epigenetic regulation of DNRR (after (Jing et al., 2020); modified): ULT1, which can associate with PcG protein EMF1, suppresses DNRR by negative regulation of *ERF109* that is activated by the wounding-induced Jasmonate signaling (Zhang et al., 2019; Tian et al., 2022). ERF109 and ERF111 activate the auxin synthesis gene *ASA1* and the transcription factor SPL10 that in turn represses *ASA1* (Ye et al., 2020). Auxin synthesis and transport produce an auxin maximum near the wounding site, which triggers the GRN composed of *ARFs*, *WOX11/12*, *LBD16/18*, *WOX5/7* and *PLT1/2*. (2) Pluripotency acquisition in the middle cell layer of callus: WOX5 and PLT1/2 directly interact to activate *TAA1* to accelerate endogenous auxin production. WOX5 also interacts with the B-type ARABIDOPSIS RESPONSE REGULATOR12 (ARR12), which represses A-type ARRs breaking the negative feedback loop in cytokinin signaling. Overall, the promotion of auxin biosynthesis and the enhancement of cytokinin sensitivity are both required for pluripotency acquisition for shoot and root regeneration (Zhai and Xu, 2021). (3) Pluripotency acquisition in microcallus: Protoplast isolation from differentiated mesophyll cells induces stochastic gene expression of *WUS* and *DORNRÖSCHEN/ENHANCER OF SHOOT REGENERATION1* (*DRN/ESR1*), which accelerate microcallus formation and, subsequently, shoot regeneration (Xu et al., 2021). The stochastic gene expression of *WUS* and *DRN* in protoplasts and microcalli might be caused by variant levels of epigenetic modifications such as H3K27me3 by PRC2. **(C)** Models of cell-type-specific transcriptional and epigenetic regulation, interaction and subnuclear localization during distal root SCN maintenance. WOX5 represses QC divisions by repressing *CYCLIN D* (*CYCD*) activity within the QC (Forzani et al., 2014). Although WOX5 and PLT2/3 proteins are present in nuclei of QC and columella stem-cells (CSCs), PLT3 recruits WOX5 only in the CSCs into nuclear bodies. The H3K27me3-reader PWWP-DOMAIN INTERACTOR OF POLYCOMBS1 (PWO1) associates with PRC2 and can form nuclear PcG bodies (Hohenstatt et al., 2018; Mikulski et al., 2019). Since TPL can recruit PRC2 activity, WOX5-TPL-HDA19 and PWO1-PRC2 might co-localize in the same nuclear body. In the columella cells (CCs), CDF4 is expressed promoting cell division und differentiation. **(B, C)** In future studies, it would be interesting to address whether WUS, WOX5 and other pluripotency factors interact with further epigenetic regulators. Arrows, transcriptional activation. Arrows with blunt ends represent repression. Double-sided arrows, protein-protein interaction. Dotted arrows indicate either indirect regulation or possible direct regulation that is not yet verified. Arrows in green indicate positive epigenetic regulation, in red, negative epigenetic regulation, and in black, non-epigenetic regulation. K4m, H3K4me3; K27m, H3K27me3; K36m, H3K36me3; H3ac, H3 acetylation; H3/4ac, H3/H4 acetylation.

Shoot branching requires the initiation of auxiliary meristems (AMs) and *de novo* stem-cell formation from a few cells of the leaf axil that retain some meristematic characteristics, named premeristems (Nicolas and Laufs, 2022). In the premeristemic cells, ATH1-STM heterodimer binds the pluripotency gene *STM* to maintain its expression at a low level, which endows permissive *STM* chromatin for subsequent upregulation of *STM* during AM initiation (**Figure 2A**). During the premeristemic stage, low *STM* transcription maintains low levels of repressive H3K27me3 and high levels of the active chromatin marker H3 acetylation, which allows the *STM* locus to remain epigenetically active (Cao et al., 2020). Hence, pluripotency seems sustained through low and epigenetically maintained expression of pluripotency genes in precursor-cells of stem-cells. It would be certainly interesting to investigate whether this is true throughout the plant life-cycle demonstrating that pluripotency is epigenetically maintained in plant stem-cell lineages.

## Stem-cell determinacy during flower development by epigenetic silencing of several pluripotency genes

Flowers are determinate structures. A feedback loop, built by *WUS* and *AGAMOUS* (*AG*), terminates the stem-cell pool of FMs by silencing *WUS* during floral stage 6, which is time-buffered *via* epigenetic regulation by PcG and TrxG proteins (Müller and Goodrich, 2011). In the last two decades, near a dozen pathways were discovered, which also contribute to the epigenetic silencing of *WUS* by H3K27me3 (**Figure 1E**). We recently revealed that during FM arrest also several other pluripotency genes, including *STM* and other TALE *KNOX* genes, are silenced by H3K27me3 (**Figure 1F**). This synchronized silencing of several pluripotency genes could accelerate FM determinacy in a way that cannot be achieved by silencing *WUS* alone (Müller-Xing et al., 2022). Considering the complexity of the gene regulatory network (GRN) controlling H3K27me3-mediated silencing of *WUS* (**Figure 1D**), similar complex regulation might wait to be revealed for the other H3K27me3-silenced floral pluripotency genes.

## Epigenetics facilitate *de novo* organogenesis from vascular-associated pluripotent cells

Similar to shoot branching, *de novo* organogenesis depends on *de novo* stem-cell regeneration from pluripotent precursor-cells. *De novo* root regeneration (DNRR) can occur directly from detached organs such as leaves (Chen et al., 2014) or indirectly, like shoot regeneration, from auxin-induced callus tissue (Skoog and Miller, 1957). Lateral roots (LRs), adventitious roots (ARs) and callus derivate from pluripotent cells that can be collectively termed vasculature-associated pluripotent cells (VPCs): LRs rise from pericycle cells (Malamy and Benfey, 1997), the root-founder cells of ARs emerge through cell-fate-transition from procambium cells or its nearby parenchyma cells (Liu et al., 2014), and callus origins from xylem-pole pericycle and pericycle-like cells (**Figure 2B**) (Atta et al., 2009; Sugimoto et al., 2010). The formation of LRs, ARs and callus is regulated by several epigenetic regulators including PRC2 (Reviewed in (Jing et al., 2020). Callus does not consist of dedifferentiated cells (Fehér, 2019) and comprise rather features of LRPs including stem-like cells. Treatment on shoot or root induction medium (SIM or RIM) gives raise to *de novo* organogenesis (**Figure 2B**). A recent study using single-cell transcriptomics revealed that only the middle cell layer of callus acquires pluripotency, which is required for organ regeneration (Zhai and Xu, 2021). In this QC-like middle layer, pluripotency depends on WOX5 that functions as master regulator activating auxin synthesis while suppressing cytokinin signaling (**Figure 2B**). The pluripotency acquisition of callus depends also on histone modifications and nucleosome assembly, since overexpression of the atypical histone variant H3.15, which cannot be H3K27me3, promotes callus formation (Yan et al., 2020). During shoot regeneration, *WUS* is essential for *de novo* establishment of the shoot SCN. After transferring the callus to SIM, *WUS* is epigenetically reactivated by a two-step mechanism: (i) the cytokinin-rich environment initially promotes the removal of repressive H3K27me3 at *WUS* in a cell cycle-dependent manner; subsequently (ii), B-type ARABIDOPSIS RESPONSE REGULATORs (ARRs) spatially activate *WUS* expression through binding with HD-ZIP III transcription factor REVOLUTA (REV) (Zhang et al., 2017). With extreme treatments, single somatic cells also can reacquire pluripotency and totipotency forming into entire plants (Takebe et al., 1971). While WOX5 provides pluripotency in auxin-induced callus, WUS accelerates microcalli formation and, subsequently, shoot regeneration from mesophyll protoplasts (Xu et al., 2021). It is yet not known whether WUS and WOX5 interact with epigenetic regulators to provide pluripotency during *de novo* organogenesis (**Figure 2B**).

## Discussion

In the last 15 years, numerous studies have enhanced our understanding about how epigenetics play a crucial role in initiation, maintenance and determination of plant stem-cells, yet many open questions remain. For example, the HIRA complex deposits the histone variant H3.3 that correlates with active gene transcription (Nie et al., 2014). However, HIRA interacts with AS1 and AS2 to repress the class I *KNOX* genes (Phelps-Durr et al., 2005). HIRA has at least the potential to act in gene repression since HIRA contributes to nucleosome occupancy also at heterochromatic regions that are silenced (Duc et al., 2015). Similarly, TrxG factors ATX1 and ULTRAPETALA1 (ULT1) and the PcG protein EMF1 interact to prevent synergistically seed gene misexpression in RAMs (Xu et al., 2018). Yet the exact molecular mechanisms remain unclear how epigenetic regulators such as HIRA and ATX1/ULT1, whose main function is transcriptional activation, achieve gene repression. In general, the question remains how the epigenetic pathways corporate with each other and which upstream signals modulate their spatiotemporal specificity. Most recently, it has been shown that glucose-activated TOR kinase controls genome-wide H3K27me3, which limits SAM size (Ye et al., 2022) indicating that glucose signaling controls stem-cell fate also epigenetically.

Some findings of single-gene approaches were recently challenged by whole-genome studies. For example, *STM* misexpression in PRC1 mutants suggested that *STM* is a direct target (Xu and Shen, 2008), while recent ChIP-Seq data show that *STM* is an H3K27me3-target but not of the PRC1 mark H2AK121ub (Zhou et al., 2017). Similarly, manipulation of cell cycles with pharmacological agents suggests that *KNU* loses H3K27me3 passively by cell division in flower primordia (Sun et al., 2014), while ChIP-Seq data show that demethylases RELATIVE OF EARLY FLOWERING6 (REF6), ELF6 and JMJ13 are required for active H3K27me3-removal at *KNU* in inflorescences (Yan et al., 2018). It should be emphasized that passive and active H3K27me3-removal could work hand-in-hand at the *KNU* gene locus.

A recent study showed that the pluripotency factors WOX5, PLT2 and PLT3 form alternative complexes in different cell-types of the RAM (Burkart et al., 2022). It seems reasonable to anticipate that epigenetic complexes and their recruiters are also cell-type-specifically formed (**Figure 2C**). Although single-cell studies are currently rather used for whole-genome profiling, cell type-specific investigation will also become more common in single-gene/protein approaches in which protein-protein and protein-DNA interactions are investigated. Advanced confocal microscope approaches reached now a new level that allows *in vivo* FRET-FLIM in single cells of the RAM (Long et al., 2017), which will reveal cell-type-specific protein interactions of epigenetic complexes in the future.

## Data availability statement

The original contributions presented in the study are included in the article. Further inquiries can be directed to the corresponding author.

## Author contributions

RM-X conceived, illustrated, and wrote the manuscript with the help of QX. All authors contributed to the article and approved the submitted version.

## Funding

This work was kindly funded by the National Natural Science Foundation of China (project nos 31640054 and 31771602), and Starting Grants of the Lushan Botanical Garden, Chinese Academy of Sciences, Jiujiang (project nos 2021ZWZX24 and 2021ZWZX25).

## Acknowledgments

We thank Danyu Kong and Xiaokun Liu for critical reading and comments on the manuscript. We would like to apologize for mostly focusing on histone H3 modifications and H3 variants in *Arabidopsis thaliana* and not covering many fundamental original studies due to space limitations. For a broader and deeper insight to the field of epigenetic regulation of plant stem-cells, we recommend the review by (Singh et al., 2020) to the readers.

## Conflict of interest

The authors declare that the research was conducted in the absence of any commercial or financial relationships that could be construed as a potential conflict of interest.

## Publisher’s note

All claims expressed in this article are solely those of the authors and do not necessarily represent those of their affiliated organizations, or those of the publisher, the editors and the reviewers. Any product that may be evaluated in this article, or claim that may be made by its manufacturer, is not guaranteed or endorsed by the publisher.
